# The Practical Application of the Roentgen Rays in Therapeutics and Diagnosis

**Published:** 1903-09

**Authors:** 


THE PRACTICAL APPLICATION OF THE ROENTGEN RAYS IN THERAPEUTICS AND DIAGNOSIS. By WILLIAM ALLEN PUSEY, A. M., M. D., Professor of Dermatology in the University of Illinois; and Eugene W. Caldwell, B. S., Director of the Edward N. Gibbs *x*-Ray Memorial Laboratory of the University and Bellevue Hospital Medical College, New York. Philadelphia, New York, London: W. B. Saunders & Co. 1903. (Cloth, $4.50 net; sheep or half-morocco, $5.50 net.)

Complicated instruments are coming to be more and more employed in medicine, and the importance of a thorough training in physics is emphasised by the now popular use of the *x*-ray outfit.

The authors of this volume have realised the importance of such training and have devoted 215 pages of their work to equipment, giving the essentials of *x*-ray tubes, induction coils, static machines and their management, fluoroscopes, radiography and photography. While this is done in the most scientific way, nevertheless, one is at a loss to determine details when using the apparatus for a specific disease.

For the second part, consisting of 315 pages by Pusey, we have nothing but praise to accord. The author wisely sets down his personal experience, which cannot be otherwise than extremely valuable, considering the fact that he has come to be regarded as the pioneer expert in this branch of medicine.

The reviewer has listened to his discussions for a number of years and is in full sympathy with his views and purposes. Dr. Pusey is not a mere experimenter; he is a thoroughly scientific and rapidly-growing man. His abundant and fruitful experience has specially fitted him for the preparation of his part of the book, for adept teaching must necessarily be based upon personal observations.

The literature bearing upon the subject has also been carefully looked after. The author has been able to do this part well, although, undoubtedly, it was a hard task on account of so many conflicting statements relating to the effects of the *x*-ray in connection with therapeutic work. He is satisfied that the *x*-ray will cure the majority of maligant growths of the cutaneous surface, claiming that this fact is not merely established clinically, but microscopically. It is painless; it leaves fewer scars; the cosmetic effect is much more satisfactory. But after all, it is a question in the reviewer's mind whether we should abandon our old methods in a large per cent of cases. Even the cases quoted by Dr. Pusey could, undoubtedly, have been successfully treated otherwise than as he directs.

The *x*-ray is "nuts" for the charlatan, who so much depends upon awing his patient and impressing him with a sense of the supernatural. This work of Drs. Pusey and Caldwell will at once become the standard authority in connection with *x*-ray treatment. It is far and away ahead of anything previously given to the profession and will be appreciated by it as a ne plus ultra.

